# Overestimation of stereo thresholds by the TNO stereotest is not due to global stereopsis

**DOI:** 10.1111/opo.12371

**Published:** 2017-03-23

**Authors:** Kathleen Vancleef, Jenny C. A. Read, William Herbert, Nicola Goodship, Maeve Woodhouse, Ignacio Serrano‐Pedraza

**Affiliations:** ^1^ Institute of Neuroscience Newcastle University Newcastle‐upon‐Tyne UK; ^2^ Faculty of Psychology Complutense University of Madrid Madrid Spain

**Keywords:** randot stereotest, global stereopsis, local stereopsis, random dot stereogram, stereo threshold, TNO stereotest

## Abstract

**Purpose:**

It has been repeatedly shown that the TNO stereotest overestimates stereo threshold compared to other clinical stereotests. In the current study, we test whether this overestimation can be attributed to a distinction between ‘global’ (or ‘cyclopean’) and ‘local’ (feature or contour‐based) stereopsis.

**Methods:**

We compared stereo thresholds of a global (TNO) and a local clinical stereotest (Randot Circles). In addition, a global and a local psychophysical stereotest were added to the design. One hundred and forty‐nine children between 4 and 16 years old were included in the study.

**Results:**

Stereo threshold estimates with TNO were a factor of two higher than with any of the other stereotests. No significant differences were found between the other tests. Bland‐Altman analyses also indicated low agreement between TNO and the other stereotests, especially for higher stereo threshold estimates. Simulations indicated that the TNO test protocol and test disparities can account for part of this effect.

**Discussion:**

The results indicate that the global – local distinction is an unlikely explanation for the overestimated thresholds of TNO. Test protocol and disparities are one contributing factor. Potential additional factors include the nature of the task (TNO requires depth discrimination rather than detection) and the use of anaglyph red/green 3D glasses rather than polarizing filters, which may reduce binocular fusion.

## Introduction

Normal stereoscopic vision is associated with correct development of visual functions and alignment of the eyes.^1^, ^2^, ^3^ Measuring near stereopsis is therefore common in children with suspected amblyopia or strabismus. Abnormalities in stereopsis are typically used to inform diagnosis and decision‐making in treatment.^2^, ^4^, ^5^

There are several clinical stereotests available on the market. A recent survey we conducted among eye health care professionals in the UK, US and Canada indicated that the most commonly used tests are Frisby (39%, Frisby Stereotests™ http://frisbystereotest.co.uk/), TNO (19%, Lameris http://www.ootech.nl/), Titmus Fly and Circles (16%; Stereo Optical Company http://www.stereooptical.com/), Randot Stereotest (12%; Stereo Optical Company http://www.stereooptical.com/), Preschool Randot Stereotest (7%; Stereo Optical Company http://www.stereooptical.com/) and Lang (6%, Lang‐Stereotest http://www.lang-stereotest.com/; Vancleef K. and Read, J. C. A., unpublished data). Previous studies have compared stereo thresholds obtained with different clinical methods. They have shown that stereo thresholds obtained with TNO are on average higher than thresholds obtained with other methods in a normal adult population^6^, ^7^, ^8^ and in patients with impaired binocular vision.^8^, ^9^, ^10^, ^11^, ^12^

One potential reason for the discrepancy in results between different stereotests relates to the distinction between global and local stereopsis.^6^, ^7^, ^9^ Global stereopsis (or cyclopean stereopsis) is measured with random dot stereograms like TNO or Preschool Randot.^13^ These stereograms do not (ideally) contain monocular cues to the objects they depict. Rather, form detection and object recognition follow the extraction of disparity by a process resembling local cross‐correlation of the left and right image. This process depends on disparity‐selective neurons in primary visual cortex,^14^ in contrast to local stereopsis which appear to have other neural substrates.^15^, ^16^, ^17^, ^18^ These neurons allow very precise, fine stereopsis, but only over a narrow fusional range.^14^, ^19^ Therefore, global stereopsis requires adequate motor alignment of the eyes, which is harder to achieve without monocular cues.^15^, ^20^

Local stereopsis (or contour stereopsis) is measured with contour stereograms like the circles and animals in the Randot stereotest or the circles and fly in Titmus. These stimuli have high‐contrast monocularly‐visible contours which can aid stereopsis in two ways. First, they provide a stronger cue to vergence, making it easier to achieve the correct motor alignment.^20^ Second, even if vergence is not correct, so the stimuli have an unfusibly large disparity on the retina, qualitative depth judgments can still be made.^21^ This is not possible with dense random‐dot stereograms, where unfusible disparities do not result in any depth percept.^22^ Perhaps for this reason, several authors have suggested that local stereopsis may be spared more often than global in binocular vision disorders like amblyopia and strabismus.^6^, ^15^, ^23^, ^24^, ^25^ More seriously, contour stereograms also have monocular displacement cues which potentially make it possible to solve the task with one eye.^23^, ^26^, ^27^

It has been suggested that the higher stereo thresholds measured with TNO are due to the presence of monocular cues in the comparing tests,^6^, ^28^, ^29^ the difficulty of global stereopsis tests compared to local stereopsis tests^6^, ^7^, ^28^ and the requirement of perfect motor alignment of the eyes.^7^, ^9^ All these explanations are related to the distinction between global and local stereopsis, explanations that have not yet been tested.

In the current study, we explore whether the distinction between global and local stereopsis can provide an explanation for the higher stereo thresholds measured with TNO compared to other stereotests. As well as clinical stereotests (TNO and Randot Circles), we used two computerised psychophysical experiments. In these psychophysical tasks, an adaptive staircase procedure and wide range of disparity levels can be used, making it possible to accurately measure stereo thresholds with a small number of trials.

## Methods

### Participants

One hundred and fifty‐three children took part in the study. Four children were unable to record a measurable threshold on any tests they tried, and were excluded from subsequent analysis. The remaining 149 participants were aged between 4.4 and 16.3 years (mean age 9.3 years, S.D. = 2.4, unreported age for six participants). Ten of these 149 participants failed to record a threshold on at least one of the tests despite passing another; we discuss below how we analysed these data. Seventy‐four participants were female and 71 were male; gender was not recorded for the remaining four participants. All of the participants were recruited at a local science museum, Centre for Life (http://www.life.org.uk). Because we aimed to study stereovision in the general population, no children were excluded based on eye pathology, but they were asked to wear their habitual correction. Parents or other accompanying adults provided informed written consent for the child. The study was approved by the Newcastle University Faculty of Medical Sciences Ethics Committee and fulfilled the tenets of the Declaration of Helsinki.

### Design

**Figure 1:**
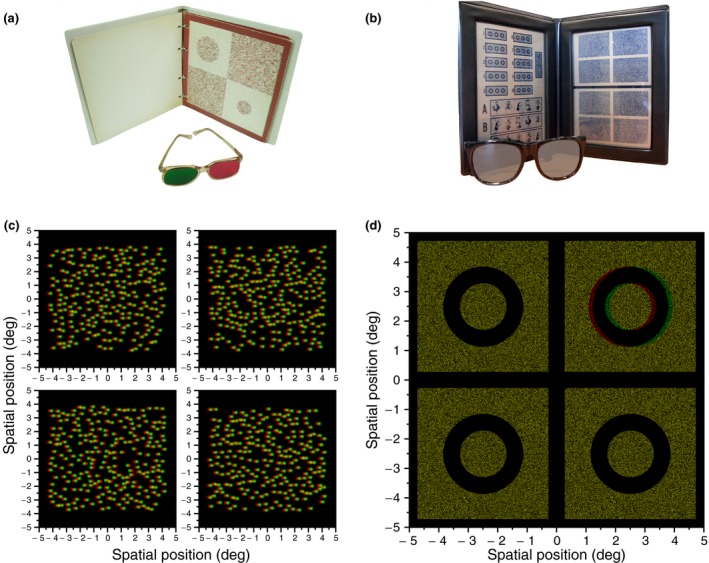
The top row (panel a and b) show the clinical tests, while the bottom row (panel c and d) shows the stimuli of the psychophysical tests. The tests shown at the left (a, c) make use of random dot stereograms to measure global stereopsis, while contour stereograms for local stereopsis are presented at the right (b and d). (a) Screening page of TNO. (b) Randot Circles are shown at the top of the left page. The Animals used for screening and familiarisation are shown at the bottom of the left page. (c) Anaglyph version of the stimuli in the Global Psychophysical test. (d) Anaglyph version of the stimuli in the Local Psychophysical test (the red filter should be placed in front of the left eye).

All children completed at least two out of the four stereotests described below (*Figure* 1). We quantified stereo threshold with two clinical tests (TNO and Randot Circles) and in two psychophysical tests (Global and Local Psychophysical Test). One of each measured global stereopsis through random dot stereograms (TNO and Global Psychophysical Test), the other measured local stereopsis through contour stereograms (Randot Circles and Local Psychophysical Test). The order of the tests was randomised. Data were collected at a dimmed area in the museum with luminance in the photopic range.

### Stereotests

The *Randot Circles* (Stereo Optical, Inc., Chicago, USA http://www.stereooptical.com/) is a clinical local stereopsis test that was administered at 40 cm. The child was familiarised with the test and the polarized glasses through the Randot Animals. They were asked to point to the animal that seemed to float in front of the page. The animals are shown at disparities of 400, 200, and 100 arcsec. Following screening with the Randot Animals, the experimenter proceeded to the Circles. Starting with the largest disparity in a descending scale, the child was asked to point to or verbally identify the circle out of three that appeared to be floating in front of the page or jumping out of the page. Unlimited viewing time was given. Target circles were presented at the following disparity levels: 400, 200, 140, 100, 70, 50, 40, 30, 25, and 20 arcsec. If the child made a mistake, the examiner went back three disparity levels and started again from there. The final threshold was derived as the mean of the last levels that were answered correctly in both runs of the Randot Circles.^30^ Feedback was not provided.

The *TNO Stereo test* (Lameris Intrumenten, Groenekan, the Netherlands, 9th or 10th edition http://www.ootech.nl/), a clinical test for global stereopsis, was performed at a distance of 40 cm. While wearing red/green anaglyph stereo glasses, the child was first presented with Plate I in which two butterflies are presented in a random dot stereogram; one is only visible when both eyes are used. If a mistake was made, the child was guided to the correct answer by the examiner. Plate II shows four discs of which two can only be seen stereoscopically. In the last screening plate, Plate III, the child had to identify four geometric shapes. Following this screening and familiarisation phase, threshold measures were obtained using Plates V–VII. In these plates, discs with a sector missing are presented and the child is asked to point to the missing part of the pie or cake. The tested levels of disparity were: 480, 240, 120, 60, 30, and 15 arcsec. For each disparity level, two stimuli were shown for an unlimited time. The experimenter moved though the disparity levels until they reached sub‐threshold disparities; no feedback was given. Following an incorrect answer the experimenter started again from three disparity levels back. The final threshold was derived as the mean of the last levels that were answered correctly in both runs.^30^

In the G*lobal Psychophysical Test (Exp Global)*, children performed a disparity detection task in which they indicated which stimulus out of four showed a square that was standing out in depth. Subjects were presented with dynamic random‐dot stereograms consisting of bright coloured dots on a black background. Each dot was given a colour generated by selecting the R, G and B values independently from a uniform distribution between minimum and maximum luminance. The dots were generated using the Psychtoolbox's ‘Screen(“DrawDots”)’ function, specifying circles 10 pixels in diameter with high‐quality anti‐aliasing. Because the display used line interleaving, the dots appeared as ellipses on‐screen, with a width of 10 pixels and a height of 20 physical pixels (10.6 × 20.12 arcmin). The pattern of dots was updated (new random positions and colours) every frame at 60 Hz.

The target was presented on one out of four positions on the screen (see *Figure* 1 *c*). The target consisted of a square patch of dots (4.13 × 4.13°) that had a crossed disparity and was located in the centre of the background made up of a rectangle of dots (8.89 × 7.31°; *W* × *H*) with opposite disparity. Thus, target and background had equal and opposite disparity relative to the screen. In the other three positions the whole rectangle was covered by dots with the same background disparity. The stimulus disparity was defined as the relative disparity between the square target and background. The presented disparity levels were not limited to fixed values as in the clinical tests, but were chosen based on previous answers of the child following an adaptive weighted one‐up one‐down staircase. The staircase started with a practice trial at a disparity of 3 log_10_ arcsec (i.e. 1000 arcsec). In addition to the disparity a non‐stereo colour/luminance cue was added to the practice trial to ease understanding of the task (all target dots were presented in red at maximum luminance). In the subsequent trials the colour/luminance cue was removed and the stimuli could only be discriminated based on disparity. Following each correct answer, disparity was decreased by 0.15 log_10_ arcsec. Following each incorrect answer disparity was increased with three times this value or 0.45 log_10_ arcsec. The staircase targeted probability correct of 0.75. The stimulus was displayed until the child made a response. Each child completed 80 trials. No feedback was provided during the experiment.

Threshold estimates were obtained by fitting a logistic function to the data^31^:

(1) $$\psi \left(x\right)=\gamma +\frac{1-\lambda -\gamma }{1+\text{exp}[\beta (\alpha -x)]}$$

where *x* is log‐disparity, β is the slope; α is the location; γ is the guessing rate (0.25); and λ is the lapse rate defined by λ = λ*(1−γ), where λ* is the probability of lapsing, in our psychophysical experiments this value was fixed to 0.05. The maximum likelihood criterion was used to determine the best fitting psychometric function with two free parameters θ and σ defined as follows:

(2) $$\theta =\alpha -\frac{1}{\beta }\text{ln}\left[\frac{1-\lambda -\pi }{\pi -\gamma }\right]$$

(3) $$\sigma =\frac{2}{\beta }\text{ln}\left[\frac{1-\lambda -\gamma -\delta }{\delta }\right]$$

where π is the probability (π = 0.75) that corresponds to the threshold value θ (in log units); and σ is the spread of the psychometric function (with δ = 0.01 so σ = 8.504/β).

Estimates were forced to stay within the 0–3 log_10_ arcsec limits for thresholds and 0–5 log_10_ arcsec for the spread.

In the *Local Psychophysical Test (Exp Local)*, contour stereograms were used similar to the Randot Circles. *Figure* 1 *d* shows an example of the stimuli used in the experiment. Four black circles were shown on a square background filled with static white noise. Each circle had a diameter of 2.65° diameter and the square background 5°. One of these four circles was standing out in depth (the background was set to zero disparity). The disparity of the circle was adjusted following the procedure described above and responses were given in the same way as in the Global Psychophysical Test. Thresholds were estimated by fitting psychometric functions as explained in the previous paragraph.

### Apparatus

Stimuli for the psychophysical tests were presented on a 23 inch passive 3D monitor (D2367PH, AOC) with a refresh rate of 60 Hz and a spatial resolution of 1920 × 1080 pixels (52 × 29 cm). The 3D stimuli were presented using the line‐interleaved stereo mode of Psychotoolbox's Psychimaging function.^32^ Left and right images are separated by circular polarized 3D glasses (Sky). Children were seated at 90 cm from the monitor (so a pixel subtended 60.4 arcsec on average) with their head in a forehead and chin rest (UHCOTech HeadSpot, Houston, USA https://www.opt.uh.edu/research/uhcotech/headspot/). They responded via a 5‐button ResponsePixx Handheld (VPixx Technologies Inc., Montreal, Canada http://vpixx.com/) with the buttons positioned in a dice layout. The four corner buttons corresponded with the four spatial locations of the stimuli (where the target could appear), the centre button was not used in the experiment. Data were collected on a DELL workstation (Intel(R) Core(TM) i3 CPU 540 @3.07GHz, 4GB RAM, 64‐bit Operating System, Windows 7), with a GeForce GTX 460 graphics card (NVIDIA), running matlab R2012a, 64‐bit (Mathworks https://uk.mathworks.com/) and Psychophysics Toolbox extensions.^32^

### Data‐analyses

The highest disparity presented in TNO and Randot Circles is 480 and 400 arcsec respectively. Thresholds therefore could not be obtained on these tests for subjects whose stereoacuity is worse than this, or who are stereoblind. We examined two ways of dealing with this: first, excluding the 11 threshold estimates (in 10 subjects) which exceeded 500 arcsec (above 480 in TNO, above 400 in Randot Circles and above 500 in the psychophysical tests), in order to examine inter‐test agreement for subjects with measurable stereovision, and second, assigning all 11 the same notional value of 800 arcsec. All stereo thresholds were log_10_ transformed to meet the normality assumption of the linear mixed model,^33^ to express the stereothresholds from all the different tests in the same units, and to account for the variability of the differences between thresholds as the average threshold increases.^34^

To account for the variation in stereoacuity between subjects we performed a linear mixed model with a random intercept and factor Test (TNO was the reference category) on the 316 threshold estimates from 149 subjects. This was followed up by pairwise comparisons between the four stereotests. Bland‐Altman analyses^34^, ^35^ informed us about agreement of the stereotests across the entire spectrum on stereo thresholds. We determined the mean difference, the confidence interval of the mean difference, and the limits of agreement (mean difference ± 1.96 × S.D.). For each pair of stereotests, the average difference between the tests and the limits of agreement were plotted against the mean of the two stereo threshold estimates. Finally, we calculated correlations between the stereotest thresholds.

### Simulations

The two clinical tests differ not only in the stimuli but also in the number of alternatives (four alternative forced choice or 4AFC for TNO and three alternative forced choice or 3AFC for Randot Circles), the available disparities, and the testing procedure. For example, the Randot Circles test starts at 400 arcsec and after each correct response the disparity is reduced until there is an incorrect response. Then the experimenter starts again presenting plates from three disparity levels back. TNO starts with 480 arcsec, and after a correct response a second stimulus with the same disparity is presented, and only if both responses are correct a plate with reduced disparity is presented. Thus in TNO, two correct responses in a row are needed in order to present a plate with lower disparity. As in Randot, after an incorrect response the experimenter starts again but three disparities levels back. For both clinical tests, the final threshold is obtained from the mean of the disparity presented in the last correct presentation in both runs. These differences may affect the final threshold estimation, quite independent of the differences in stimuli.

**Figure 2:**
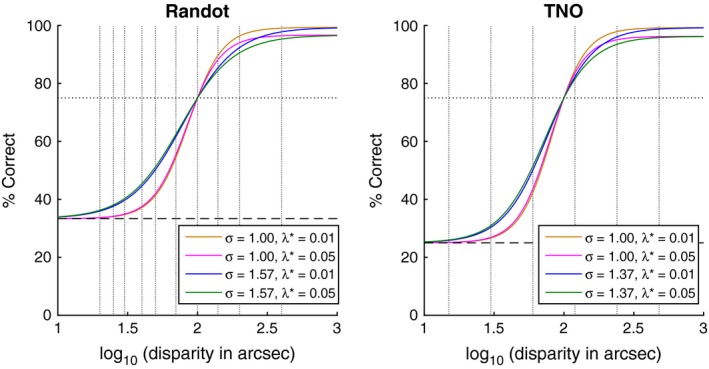
Model psychometric functions used in the simulations (coloured lines), together with the test values (vertical dotted lines). The plots show psychometric functions with a 75% threshold of 2 log_10_ arcsec (100 arcsec). For higher or lower thresholds, the functions would simply shift left or right on these axes.

We used simulations to assess the statistical properties of the Randot Circles and TNO clinical tests. We used a ‘model observer’ with the logistic psychometric function specified in *Equation* (1). We considered model observers with 12 different stereoacuities, corresponding to thresholds θ (at π = 75%) ranging from 1.4 to 2.5 log_10_ arcsec (25–320 in arcsec). The parameter δ was fixed at 0.01, the guessing rate γ was 0.25 for TNO (4AFC) and 0.33 for Randot (3AFC), and we examined different values of the lapse rate λ and spread σ (see *Table* 1). The resulting psychometric functions are shown in *Figure* 2.

**Table 1:** Parameters of the Model Logistic function used in the simulations

Clinical test	γ	δ	π	θ	λ*	σ
TNO	0.25	0.01	0.75	1.4–2.5 in steps of 0.1	0.01 or 0.05	1 or 1.37
Randot	0.33	0.01	0.75	1.4–2.5 in steps of 0.1	0.01 or 0.05	1 or 1.57

We simulated 20 000 threshold estimations for each model observer. For each threshold estimation, we ran the clinical tests exactly as with human observers, except that the response of the subject was replaced by a pseudorandom binary number generator in which the probability of a correct response was read off from the model observer's psychometric function evaluated at the disparity presented in the trial.

## Results

### Stereothresholds from human participants

We observed an average threshold estimate of 1.88 log_10_ arcsec for TNO (S.D. = 0.41 log_10_ arcsec). The average threshold estimates for the other tests were considerably lower than TNO and very similar to each other: the average threshold estimate was 1.57 log_10_ arcsec (S.D. = 0.26 log_10_ arcsec) for Randot Circles, 1.58 log_10_ arcsec (S.D. = 0.27 log_10_ arcsec) for both the Global and Local Psychophysical test.

The fitted linear mixed model had an intercept of 1.89 log_10_ arcsec; this corresponded to the average estimated threshold for TNO. For Randot Circles, the Global Psychophysical test, and the Local Psychophysical test, the estimates were −0.32, −0.29, and −0.29 respectively. These are the amounts by which the average estimated thresholds for these tests are lower than for TNO. Multiple comparisons with Tukey correction showed significant differences in estimated thresholds between TNO and the other stereotests (TNO vs Randot Circles: *z* = −8.56, *p* < 0.001; TNO vs Global Psychophysical test: *z* = −5.53, *p* < 0.001; TNO vs Local Psychophysical test: *z* = −5.93, *p* < 0.001). The threshold estimates from the other tests did not differ significantly (Randot Circles vs Global Psychophysical test: *z* = 0.52, *p* = 0.95; Randot Circles vs Local Psychophysical test: *z* = 0.54, *p* = 0.95; Global vs Local Psychophysical test: *z* = −0.01, *p* = 1). In sum, our linear mixed model confirms that the TNO produces significantly higher estimates of stereo threshold, while there is no difference in stereo threshold estimates between the other three stereotests.

**Figure 3:**
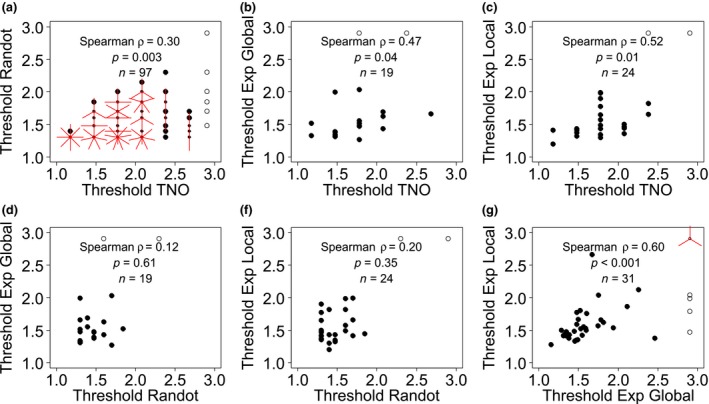
Scatterplots showing stereothresholds for all combinations of stereotests. All thresholds are given in log_10_ arcsec. ‘Exp’ refers to the psychophysical experiments. Where results from both tests are quantized, points can coincide; the number of rays from a point indicates the number of results coinciding. Open symbols represent stereoblind subjects (>500 arcsec on one of the two stereotests), who were not included in the correlations reported in the figure.

**Figure 4:**
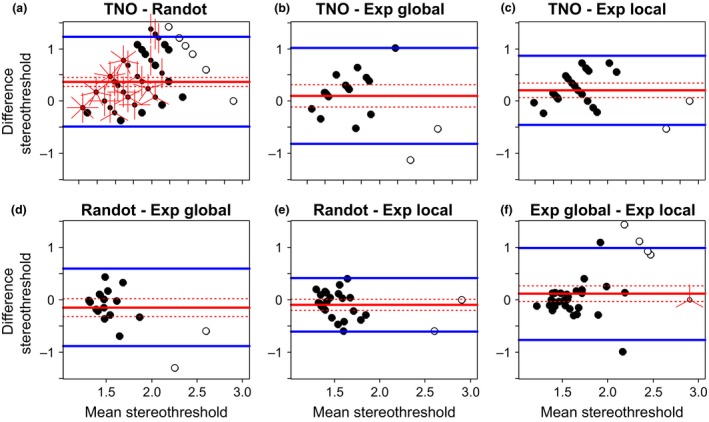
Bland‐Altman plots. Each plot shows the difference between the estimated stereo threshold (in log_10_ arcsec) of two stereotests as a function of the average estimated stereo threshold of the two stereotests. The solid red line shows the average difference with its confidence interval (dotted red lines). The blue lines shows the limits of agreement. ‘Exp’ refers to the psychophysical experiments. Where results from both tests are quantized, points can coincide; the number of rays from a point indicates the number of results coinciding. Open symbols represent stereoblind subjects (>500 arcsec on one of the two stereotests) that were not included in the main analyses.

Scatterplots for all combinations of stereotests are shown in *Figure* 3, while *Figure* 4 shows the corresponding Bland‐Altman plot. For two tests to agree well, we require *both* that the results are correlated, *and* that the mean difference and limits of agreement are small. If two tests are correlated but have non‐zero mean difference and/or wide limits of agreement, they may be giving answers that differ by a constant offset or gain. If two tests have zero mean difference and narrow limits of agreement but are not correlated, then the tests are giving nearly the same result for everyone tested, so are not informative. We therefore compare all three metrics.

On the two psychophysical experiments, results were correlated (*Figure* 3 *f*:* n* = 31, Spearman ρ = 0.60, *p* < 0.001) over a wide range of stereoacuity (thresholds ranging from 1.16 to 2.66 log_10_ arcsec). Additionally, the mean difference was near zero (−0.001, 95% CI = [−0.12, 0.12]) and the limits of agreement were relatively narrow (±0.62 log_10_ threshold, or a factor of 4). Thus, these two different psychophysical tasks give fairly similar ratings of stereoacuity.

We did not observe a correlation between threshold estimates on Randot Circles and the Global (*Figure* 3 *d*:* n* = 19, ρ = 0.12, *p* = 0.61) or Local Psychophysical tests (*Figure* 3 *e*:* n* = 24, ρ = 0.20, *p* = 0.35). This was not simply due to the lower range of stereo thresholds recorded in the subjects who performed these tasks (1.20–1.99 log_10_ arcsec), since the correlation between the two psychophysical tasks was still significant when we considered only data‐points within this range (*Figure* 3 *f*: subset *n* = 28, ρ = 0.53, *p* = 0.004). However, there was again no systematic difference (mean differences of −0.06 log_10_ arcsec, 95% CI = [−0.19, 0.06]; and −0.08, 95% CI = [−0.18, 0.03] for Randot Circles vs Global and vs Local Psychophysical tests respectively), and the limits of agreement were similar to the Local vs Global Psychophysical tasks. Thus, the Randot Circles test does not agree very well with either of our psychophysics tasks, but at least does not suffer from a bias.

**Figure 5:**
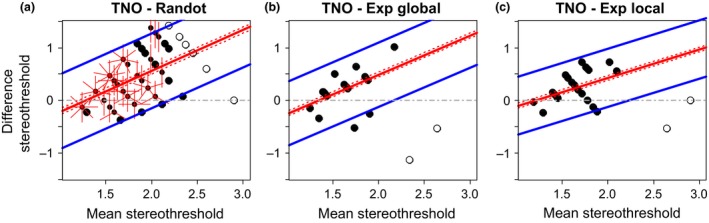
Bland‐Altman plot for agreement between TNO and other stereotests. The plots show the difference between the estimated stereo threshold (in log_10_ arcsec) of TNO and the other stereotests (Randot Circles in a, Global psychophysical test in b, and Local psychophysical test in c) as a function of the average estimated stereo threshold of the two stereotests. The solid red line shows the average difference with its 95% confidence interval (dotted red lines) determined by regressing the difference between the stereotests on the mean thresholds of both methods. The blue lines shows the limits of agreement. Where results from both tests coincide; the number of rays from a point indicates the number of results coinciding. Open symbols represent stereoblind subjects (>500 arcsec on one of the two stereotests) that were not included in the main analyses.

Thresholds on TNO and Randot Circles were only weakly correlated (*Figure* 3 *a*: ρ = 0.30), although this reached significance due to the large numbers of subjects who performed both these tests (*n* = 97, *p* = 0.003). The mean difference was 0.34 log_10_ arcsec (95% CI = [0.25, 0.42]) indicating that TNO significantly overestimates the threshold compared to Randot Circles. The limits of agreement are −0.48 and 1.15 log_10_ arcsec which reflect a large variation in agreement over the range of stereo thresholds. To account for this relationship we modelled the change in thresholds difference as a function of the mean threshold through linear regression (*F*_1,95_ = 30.36, *p* < 0.001; estimated intercept = −0.99, *t*_95_ = −4.01, *p* < 0.001; estimated slope = 0.77, *t*_95_ = 5.51, *p* < 0.001). The limits of agreement were calculated by adding and subtracting 1.96 standard deviations (S.D. = 0.36) to or from the intercept of the regression line. The agreement systematically varies across the range of stereo thresholds following the following regression equation above (*Figure* 5 *a*) with better agreement at lower mean stereo thresholds, and poorer agreement with increasing mean stereo thresholds. Thus, TNO and Randot Circles do not agree well: they are poorly correlated, and show systematic differences.

The TNO thresholds correlated with thresholds on both psychophysical tasks (Exp Global, *Figure* 3 *b*: ρ = 0.47, *n* = 19, *p* = 0.04, and Exp Local, *Figure* 3 *c*: ρ = 0.52, *n* = 24, *p* = 0.01). However, the Bland‐Altman analysis indicated that this correlation again concealed systematic differences. Agreement between TNO and the Global Psychophysical test is 0.20 log_10_ arcsec (95% CI = [0.03, 0.37]) with limits of agreement at −0.49 and 0.90 log_10_ arcsec, indicating poor agreement. Agreement seems to decrease with increasing mean threshold estimates (*Figure* 5 *b*,* F*_1,17_ = 5.34, *p* = 0.03; estimated intercept = −1, *t*_17_ = −1.90, *p* = 0.07; estimated slope = 0.74, *t*_17_ = 2.31, *p* = 0.03). Likewise, agreement between TNO and the Local Psychophysical test was low with a mean difference of 0.25 log_10_ arcsec (95% CI = [0.12, 0.38]) and limits of agreement at −0.361 and 0.86 log_10_ arcsec. Again, this seems to differ over the whole range of stereo threshold estimates (*Figure* 5 *c*,* F*_1,22_ = 4.65, *p* = 0.04; estimated intercept = −0.65, *t*_22_ = −1.54, *p* = 0.14; estimated slope = 0.54, *t*_22_ = 2.16, *p* = 0.04).

We have repeated the analyses including the thresholds above 500 arcsec but set to a notional value of 800 arcsec (since values above 500 arcsec are not available on the clinical tests). The choice of 800 arcsec is necessarily arbitrary but was chosen as being roughly midway between someone who only just failed the test (e.g. true threshold 510) and someone who has no stereovision (would fail even at 1800). We achieved the same results except for the Bland‐Altman analysis of TNO vs the Global Psychophysical test. With inclusion of the outliers, the variance increases, which means the average stereothresholds with TNO were no longer significantly different from the average stereothresholds with the Global Psychophysical test (mean difference = 0.10 log_10_ arcsec, 95% CI = [−0.11, 0.32]). With inspection of *Figures* 3 *b and* 4 *b* (open symbols) it is clear that this is due to poor agreement between the TNO and the Global Psychophysical test: the outliers achieve a high threshold on the Global Psychophysical test and a low threshold on TNO. As before, we therefore conclude there is poor agreement between both tests.

### Statistical properties of clinical stereotests

**Figure 6:**
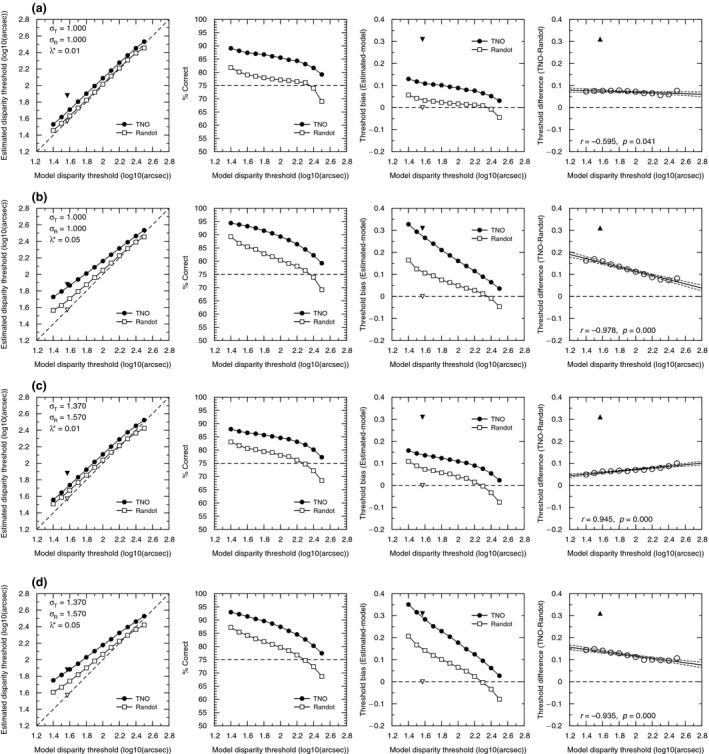
Simulation results. The values of the parameters tested (see also *Table* 1, *Figure* 2) are presented in the upper‐left corner of the panels of the left column. (a) Results for r_T_ = 1.000, r_R_ = 1.000, and a lapse rate (k*) of 0.01. (b) Results for r_T_ = 1.000, r_R_ = 1.000, and a lapse rate (k*) of 0.05. (c) Results for r_T_ = 1.370, r_R_ = 1.570, and a lapse rate (k*) of 0.01. (d) Results for r_T_ = 1.370, r_R_ = 1.570, and a lapse rate (k*) of 0.05. First column: mean of 20 000 estimated thresholds as a function of the model disparity thresholds. Error bars are omitted for clarity; the S.D. is usually ±0.2 log_10_ units. Black/white triangles correspond to the mean empirical thresholds obtained with TNO/Randot Circles (1.88 and 1.57 respectively). Their *x*‐coordinate is the value (1.58) which was obtained with both the Global and Local Psychophysical tests. Second column: Value of the model psychometric function at the estimated threshold of the stereotest. Horizontal line marks 75%, which is – by definition – the value of the model psychometric function at the disparity on the *x*‐axis. Third column: Bias of the estimated threshold (Estimated threshold – true model 75% threshold). Triangles correspond to the mean difference between the empirical thresholds for TNO (black) and Randot (white) compared with the psychophysical tests. Fourth column: difference between the estimated thresholds for TNO minus the estimated thresholds for Randot. Black triangle shows the difference of the stereothresholds obtained empirically. Zero means no difference between tests. Line represents the regression line and the 95% CI. In the bottom‐left, the values of the Pearson correlation and the associated *p*‐value are presented.

The results are plotted in *Figure* 6. The four rows of *Figure* 6 show the four different parameter‐sets we used in the simulations: two spread (σ) values and two lapse rates (λ*). In *Figure* 6 *a,b* we used the same spread value for both clinical tests (σ = 1), and examined different lapse rates: (λ*) = 0.01 in *Figure* 6 *a* and (λ*) = 0.05 in *Figure* 6 *b*. Our human psychophysical experiments also provided estimates of the spread σ. The mean value was 1.37 for the global psychophysical test and 1.57 from local. In *Figure* 6 *c,d*, we therefore examine simulations which use different values of σ for the two tests: σ_T_ = 1.37 for TNO and σ_R_ = 1.57 for Randot. As before, we also examine two different lapse rates (λ* = 0.01 in *Figure* 6 *c* and λ* = 0.05 in *Figure* 6 *d*).

The first column of *Figure* 6 plots the threshold estimated from the test against the true value of the 75% threshold. For the simulated Randot, points lie on the identity line, but thresholds from the simulated TNO are systematically higher.

Is this simply because the TNO measures the threshold corresponding to a higher performance level? The second column of *Figure* 6 shows the performance level on the model psychometric function corresponding to the estimated threshold in each case. We see that part of the difference is because TNO targets a higher threshold. For observers with thresholds in the middle of the test range and low lapse rate (<1%), the Randot targets performance of around 78%, whereas TNO targets performance around 85%. However, it is also clear neither TNO nor Randot targets a fixed level of performance. They target lower performance in observers with high thresholds, for which few of the test disparities are easily visible. This difference becomes particularly important at high lapse rate.

Comparing the four rows of *Figure* 6, we see that different values of spread cause little difference in results. Lapsing rate has a bigger effect. As expected, with a higher lapse rate, threshold estimates are higher, since on some trials the model observer will give a wrong answer for stimuli which should have been clearly visible. This is especially true for low values of the true threshold, presumably since then more of the test values are above threshold and thus liable to be affected by lapses. Lapses have a more serious effect on TNO, presumably because the subject has to avoid lapsing in two successive trials in order to progress.

The final two columns of *Figure* 6 show the bias (difference between the threshold estimated by the stereotest and the model's true 75% threshold), and the difference between the estimates provided by the two stereotests. For low lapse rate, the bias is usually under 0.05 for the Randot, but around 0.1 for TNO. Thus, even in simulations where the global/local distinction and other aspects of the stimuli have no effect, TNO produces systematically higher thresholds than Randot, or than the true 75% threshold. Across a wide range of situations, TNO overestimates thresholds by at least 25%.

This effect must surely contribute to the higher stereothresholds observed with TNO. However, our data indicate it is not the sole explanation. The triangular symbols in *Figure* 6 represent the mean of empirical data from our child participants. The means for TNO and Randot differ by 0.32 log units, corresponding to a factor of 2. As the last column of *Figure* 6 clearly shows, this is substantially higher than we ever observed in our simulations. This suggests that other factors must contribute to the discrepancy between TNO and Randot thresholds.

## Discussion

We observed higher average stereo thresholds with the TNO test than with Randot Circles stereotest or with any of our psychophysical global and local stereotests. This bias was also evident from Bland‐Altman analyses, and most profound in higher threshold estimates, while good agreement was observed at lower threshold estimates. We observed no significant difference in average threshold estimates between the other tests.

Our results are in agreement with previous studies comparing performance on TNO and Randot Circles.^6^, ^23^, ^29^ Stereo threshold estimates from earlier evaluations are presented in *Table* 2. To ease comparison with our results, we have performed a paired *t*‐test on our threshold estimates in arcsec (beside the linear mixed model analysis on thresholds in log_10_ arcsec) in the subsample of our subjects who completed both tests. Our results are in line with previous studies that point to increased threshold estimates and increased variability in TNO compared to Randot Circles, although average threshold estimates differ between the samples. Our average threshold estimate of 118 arcsec with TNO in healthy children aged 4–16 years is slightly higher than reported by Simons (109.9 arcsec in children aged 2–3^29^) and significantly higher than reported by Singh *et al*.^36^ in children and adults aged 6 or older (63 arcsec, unpaired *t*‐test: *t*_125_ = 2.37, *p* = 0.02).^29^, ^36^ Average differences of 30 arcsec between various editions of the TNO have been reported before and have been related to differences in the printing process, however this has not yet been investigated for the 9th and 10th edition of the test that were used in our study.^37^ Our results are also in congruence with a study by Gadia and colleagues who showed correspondence between stereo thresholds of Randot Circles and a software‐based stereo acuity test.^38^

**Table 2:** Previously reported stereo threshold estimates for TNO and Randot Circles (Mean ± S.D. if reported in the paper)

Paper	*n*	TNO	Randot Circles	*t* a	df	*p*
Current study	97	118 (±126.1)	41.3 (±29.3)	5.95	96	<0.001
Simons (1981)^29^						
Healthy children (2–3 years old)	38	109.9	64.1			
Healthy adults	8	40.5	21.3			
Antona *et al*. (2015)^6^						
Healthy adults	54	52 (±25)	29 (±10)	6.28	106	<0.001
Adults with abnormal binocular vision	20	158 (±149)	59 (±53)	2.80	38	0.008
Singh *et al*. (2013)^36^						
Healthy controls (>6 years old)	30	63 (±21)	23.7 (±5.1)	9.97	58	<0.001
Intermittent exotropia preoperative (>6 years old)	30	94 (±79.4)	50.3 (±59.2)	2.41	58	0.019
Intermittent exotropia 3 months postoperative (>6 years old)	30	80 (±80.1)	34.2 (±36.5)	2.85	58	0.006

a Because no individual data are available for the published papers, unpaired *t*‐tests instead of paired *t*‐tests were calculated if S.D.'s were reported.

The similar thresholds obtained with our local clinical, local psychophysical and global psychophysical stereotests indicate that the higher thresholds obtained with TNO, a clinical test for global stereopsis, cannot be attributed to increased difficulty of global stereopsis compared to local stereopsis, as has been suggested before.^6^, ^7^ Rather, the poor performance on TNO must be due to some other difference between the tests. We now consider some possible explanations.

Monocular cues in the other stereo tests can be excluded as a potential explanation, because the use of a dynamic random dot display in the global psychophysical stereotest eliminated any monocular cues.^39^ Thus if this had been the explanation, thresholds would have been elevated in our global psychophysical stereotest as well.

One possible factor is the dot size: TNO uses smaller dots and higher dot density than our global psychophysical stereotest. Westheimer has argued that smaller dot size reduces stereoacuity,^40^ but Simons^29^ suggested the opposite: bigger dots on sparser displays can reduce stereoacuity. Thus there is no clear relation between dot size and stereoacuity. We did not observe a difference between the thresholds on our local and global psychophysical stereotest although the size of the background dots differed by at least a factor of 10. Thus, dot size is unlikely to account for the difference in thresholds between TNO and the other stereotests.

Another possible explanation is that the TNO is particularly sensitive to one or more factors that affect stereoacuity, such as low visual acuity^29^ or ocular misalignment. We did not measure these, but our data still enable us to draw some conclusions. Given the different sizes of the target stimuli and the different viewing distance measure, the targets in our four tests have occupied different visual angles. However, stereo thresholds were lower with the smallest stimuli (Randot Circles occupied 0.6 by 0.6° visual angle) than for the TNO, where stimuli were 8.6 by 8.6° and the missing wedge or sector had a radius of 4.3° visual angle and angle of 60°. Thus it is not clear why poor visual acuity would affect stereoacuity measured on the TNO more than on other stereotests. Similarly, although ocular misalignment would be expected to impair performance on global stereotests more than local,^1^, ^6^, ^17^, ^24^ it is not clear why it should affect the TNO more than our global psychophysical test. Additionally, if these factors were responsible, we would expect the increase in mean threshold on the TNO to be driven largely by a subgroup of people with particularly poor scores on the TNO (these would be the people with poor visual acuity/ocular misalignment). This is not observed; in fact, thresholds on the TNO are well correlated with those on our local and global psychophysical stereotests, but are shifted upwards. Additionally, previous studies which screened participants for good visual acuity and good ocular alignment also found higher scores on the TNO.^6^, ^29^ Thus, it seems unlikely that the TNO is more sensitive to visual problems than other stereotests which measure global stereopsis.

The TNO does have poor test–retest reliability.^6^ Antona *et al*.^6^ reported a difference in stereo threshold estimates of 5 arcsec between two sessions with a coefficient of repeatability (COR = 1.69 × S.D. of mean difference) of 54 arcsec. For Randot Circles the mean difference was smaller: 1 arcsec with COR of 23 arcsec as reported by Antona *et al*.^6^ and COR of 3 arcsec as reported by Leat *et al*.^30^ Poor reliability can indeed explain the higher 95% tolerance limits in the Bland‐Altman analysis. However, this cannot explain the systematic bias we observed towards higher stereo threshold estimates in TNO.

The clinical stereotests, TNO and Randot, offer only a limited number of discrete disparity levels, whereas our psychophysical stereotests can present any disparity required by the algorithm, based on the participant's responses. To test the effect of the disparity steps used in each test and also the different procedures used, we performed a detailed simulation study. We found that TNO always overestimates the disparity threshold as compared with Randot Circles (*Figure* 6), while Randot Circles is closer to the 75% threshold targeted by our psychophysical staircase procedure. Qualitatively, therefore, this has the same tendency as our results. Quantitatively, the discrepancy depends on the subject's stereoacuity and the spread of their psychometric function, and is generally worse for higher lapsing rates. However, none of the values we explored – even a high lapsing rate of 1 in 20 – could account for the factor of two difference observed empirically between TNO and the other tests. Thus, differences in test procedure contribute to the higher thresholds observed with TNO, but cannot account for them completely.

Having rejected these explanations, what can account for the poorer scores on TNO? One possibility is that the TNO stereotest places a higher cognitive load on participants.^38^ Complexity can be attributed to the stimulus: in the TNO, children have to detect an unfamiliar shape (disk with missing sector or Pacman) compared to simple circles or squares presented in our other stereotests. Alternatively, complexity could be attributed to the task instructions: in TNO children have to ‘point to the missing piece of the cake or pie’, while in the other tests children can point to the circle or square that ‘looks different’. Simons^29^ has observed better stereoacuity when the instruction of the TNO are adapted to ‘put your finger in the hole where the piece is missing’ without naming the shape of the test figure. This explanation can be tested by running the same experiments in adults. However, previously adults also recorded higher thresholds on the TNO,^6^, ^29^ making it less likely that failure to understand instructions is the only cause.

Also, the Randot Circles and the psychophysical tasks are pure forced‐choice detection tasks in which children need to detect the circle or square standing out in depth amongst three or four possible alternatives. Shape discrimination is not necessary and a disparity level can be passed by only perceiving ‘depthness’.^29^ While shape discrimination is relatively easy in the local contour stereopsis tasks and therefore unlikely to reduce threshold estimates, in the global psychophysical task detecting just ‘depthness’ without identifying the shape can surely make the task easier in the random dot display. In the TNO, just detecting ‘depthness’ is insufficient and the shape of the Pacman needs to be identified in each stimulus.^29^ We think this is a plausible explanation.

Finally, in contrast to the other tests that use polarized glasses, the plates in TNO are viewed through anaglyph 3D glasses. Simons and Elhatton^41^ showed that anaglyph glasses introduce artefacts in binocular vision testing. Yamada *et al*.^42^ specifically compared anaglyph and polarized versions of both global and local stereopsis tests. They found good agreement between both types of glasses in the screening tests for global stereopsis, Random Dot Letter E and Random Dot Butterfly. These screening tests present stimuli with a disparity between 600 and 2000 arcsec. For the tests that measure lower levels of local stereopsis (28–800 arcsec), the authors observed inferior performance with the anaglyph glasses compared to the polarized glasses. We presented only disparities below 1000 arcsec and also observed lower performance in the test that uses anaglyph glasses (TNO) compared to the tests that use polarized glasses. Similarly, Larson observed that stereoacuity is reduced by 2–34 arcsec when wearing anaglyph glasses. In addition, for their subjects with low thresholds (15 arcsec) results were similar between local and global stereopsis (TNO), while for other subjects, performance on TNO was worse than on the local stereopsis test,^43^ similar to the distinction we observed between subjects with low vs high thresholds. Although there are differences in luminous transmittance and contrast between the red and green filters,^37^ both filters produce similar luminous flux efficiency when taking into account the CIE luminous efficiency curve of the eye in photopic conditions as was shown by Varón *et al*.^10^ This means that with adequate test picture reflectance and spectral distribution of white light, the left and right images are of similar luminance.^10^ However, in suboptimal light condition and with individual variation in CIE curves,^44^ luminance and contrast imbalance between the red and green filters might possibly have been more prominent in our testing conditions. These differences in luminous transmittance could potentially have affected suppression tendencies,^45^ increasing the stereo thresholds. As suggested by an anonymous reviewer, further research studying fusion abilities with anaglyph glasses in different light conditions in subjects with known accommodative and vergence abilities will be required to evaluate this potential explanation. More fundamentally, the colour mismatch in anaglyph is itself dissociative. As was shown by Cornforth *et al*.^46^ chromatic imbalance rather that illumination imbalance reduces stereopsis, making the colour mismatch a more plausible reason for the higher thresholds we have observed.^7^

A limitation of our study is that our data are from the general population of children, potentially including children with visual problems. We have not measured visual acuity, ocular alignment or ocular mobility in our subjects. Therefore we could not exclude children with amblyopia due to strabismus or anisometropia, conditions that are known to affect stereopsis. In addition, it might have been informative to repeat TNO testing with reversed anaglyph glasses as is recommended by Simons and Elhatton.^41^ They have observed large differences (2:1 or more) between the two positions of the glasses. Potentially, we might have observed TNO thresholds that are more in line with the stereothreshold from the other tests when reversing the anaglyph glasses. Last, although this study excludes the distinction between local and global stereopsis as an explanation for higher TNO thresholds compared to the other tests, we did not evaluate the alternative explanations suggested above. Therefore the current study cannot provide an explanation for the observed effect, but is limited to excluding one explanation: global vs local stereopsis. Follow‐up studies will be required to examine the extent to which each of the possible explanations contributes to the effect.

In sum, we have confirmed that the TNO test overestimates stereo thresholds in the general child population, and have shown that this cannot be due to differences between global and local stereopsis. We have shown that the TNO protocol and test disparities contribute to the overestimation but do not fully explain it. Other likely explanations include the greater demands of the TNO task and the use of anaglyph 3D glasses.

## Disclosure

The authors report no conflicts of interest and have no proprietary interest in any of the materials mentioned in this article.
